# Microstructure and Mechanical Properties of Si_3_N_4_-Fe_3_Si Composites Prepared by Gas-Pressure Sintering

**DOI:** 10.3390/ma11071206

**Published:** 2018-07-13

**Authors:** Jiasuo Guan, Laifei Cheng, Mingxing Li

**Affiliations:** Science and Technology on Thermostructural Composite Materials Laboratory, Northwestern Polytechnical University, Xi’an 710072, Shaanxi, China; guanjs@xaaq.com (J.G.); mingxingli@mail.nwpu.edu.cn (M.L.)

**Keywords:** Fe-Si_3_N_4_, Si_3_N_4_, ceramic composite, toughening mechanism

## Abstract

Si_3_N_4_-Fe_3_Si composites were prepared using Fe-Si_3_N_4_ as the source of Fe_3_Si by gas-pressure sintering. By adding different amounts of Fe-Si_3_N_4_ into the starting powders, Si_3_N_4_-Fe_3_Si composites with various Fe_3_Si phase contents were obtained. The microstructure and mechanical properties of the composites were investigated. With the increase of Fe-Si_3_N_4_ contents, the content and particle size of Fe_3_Si both increased. When more than 60 wt. % Fe-Si_3_N_4_ were added, the abnormal growth of Fe_3_Si particles occurred and oversized Fe_3_Si particles appeared, leading to non-uniform microstructures and worse mechanical properties of the composites. It has been found that Fe_3_Si particles could toughen the composites through particle pull-out, interface debonding, crack deflection, and particle bridging. Uniform microstructure and improved mechanical properties (flexural strength of 354 MPa and fracture toughness of 8.4 MPa·m^1/2^) can be achieved for FSN40.

## 1. Introduction

Si_3_N_4_ is one of the most promising engineering ceramics with high strength, high hardness, good oxidation, and corrosion resistance—even at high temperature. It is also an irreplaceable material in modern industries [1,2,3,4]. Numerous works have been done to lower the price of Si_3_N_4_ products in order to realize its wide application. However, in some price-sensitive areas, like the Metallurgical industry, the cost of Si_3_N_4_ is still too high. To tackle this problem, a novel refractory material, ferro-silicon nitride (Fe-Si_3_N_4_) has been developed and has successfully replaced the relatively expensive Si_3_N_4_ [5,6]. Using the ferro-silicon alloy FeSi75 as the raw material, Fe-Si_3_N_4_ powder can be synthesized by direct nitridation [7,8,9] or self-propagating high-temperature synthesis [10,11,12,13] under nitrogen atmosphere. Owing to the catalytic effect of Fe, a lower nitridation temperature and higher reaction rate can be achieved [7,14,15]. FeSi75 is extensively used as a hardener and scavenger in smelting steel, so it can be easily obtained at a low price. And the synthesis process of Fe-Si_3_N_4_ is concise, cost effective, and can be operated on a large scale. The above factors lead to the low cost of Fe-Si_3_N_4_ products.

Microstructural analysis has shown that Fe-Si_3_N_4_ was mainly composed of Si_3_N_4_ (~75 wt. %) and a small amount of un-nitrided Fe_3_Si (~15 wt. %) [16,17]. With the main phase being Si_3_N_4_, Fe-Si_3_N_4_ inherits the excellent comprehensive properties of pure Si_3_N_4_. What is more, the low melting point Fe-containing phase endows Fe-Si_3_N_4_ with good sinterability which resulted from enhanced particle rearrangement and diffusion in the presence of a more liquid phase [6].

Through the reaction bonding of FeSi75 powder compact or the sintering of Fe-Si_3_N_4_, porous Fe-Si_3_N_4_ ceramics and Fe-Si_3_N_4_ based ceramic composites for the refractory application can be prepared. Several studies have been done using these methods to fabricate Fe-Si_3_N_4_ [6], Fe-Si_3_N_4_-SiC [18,19], and Fe-Si_3_N_4_-ZrO_2_ [20] refractories. Results showed that, compared with their traditional rivals, Fe-Si_3_N_4_ based refractories exhibit high thermal strength, a higher coefficient of thermal conductivity, and better thermal shock resistance [19].

As the above application of Fe-Si_3_N_4_ has attracted much attention, the potential of Fe-Si_3_N_4_, which can be regarded as Si_3_N_4_-Fe_3_Si composite ceramics, as a thermal structural material has not been researched yet. For a long time, intermetallic compounds have been added to ceramics materials to toughen the brittle ceramics [21,22,23,24]. For instance, using Ni_3_Al intermetallic compound as a second phase, an Al_2_O_3_ composite with high strength and high toughness can be prepared [25]. Toughening effects that result from intermetallic particles can be attributed to crack deflection, crack bridging, plastic deformation [26,27], and residue stress caused by a coefficient of thermal expansion (CTE) mismatch [28]. Among the component of Fe-Si_3_N_4_, not only Si_3_N_4_, but also Fe_3_Si—which is an intermetallic compound—have good thermal mechanical properties [29,30]. So, one can infer that with both of its main phases having a high performance, the mechanical properties of Fe-Si_3_N_4_ are worth studying.

In this work, Si_3_N_4_-Fe_3_Si composites that contain different amount of Fe_3_Si with high relative density are successfully prepared by gas-pressure sintering. The microstructure and mechanical properties are studied and the effect of Fe_3_Si particles on crack propagation behavior is highlighted.

## 2. Materials and Methods

**Figure 1:**
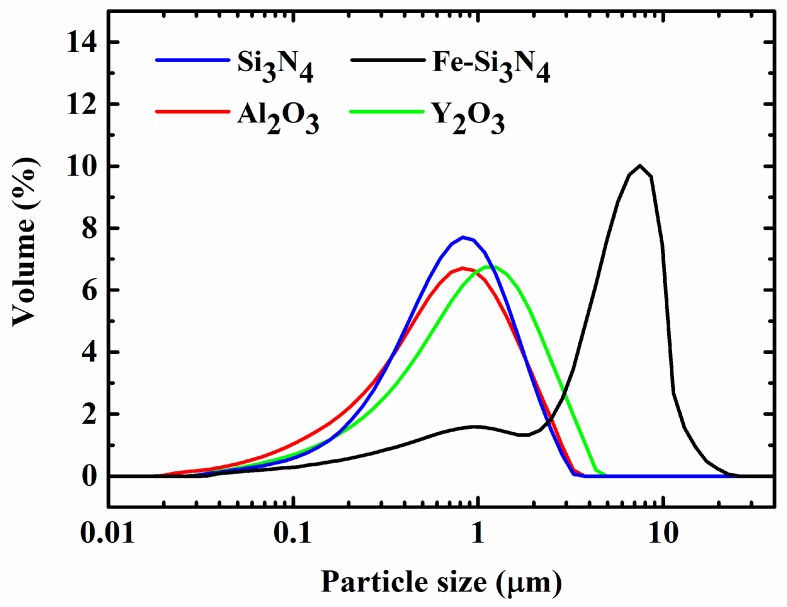
Size distribution of the starting powders.

Fe-Si_3_N_4_ powder (Fe content: 12~20 wt. %; Xi’an Aoqin new materials Co., Ltd., Xi’an, China), Si_3_N_4_ powder (purity > 99.9%, 0.8 μm, Shanghai Shuitian technology Co., LTD., Shanghai, China), Y_2_O_3_ powder (purity >99.9%, 1 μm, Shanghai Shuitian technology Co., LTD., Shanghai, China), and Al_2_O_3_ powder (purity >99.9%, 1 μm, Shanghai Shuitian technology Co., LTD., Shanghai, China) were used as starting powders. Y_2_O_3_ and Al_2_O_3_ act as sintering aids. Under high temperature, Y_2_O_3_ and Al_2_O_3_ react with SiO_2_ or silicon oxynitride, which are always present on the surfaces of Si_3_N_4_ powders, to form a liquid phase that is beneficial for densification. The size distribution of the starting powders are given in Figure 1. In order to adjust the content of Fe_3_Si in the final composites of the Si_3_N_4_-Fe_3_Si composites, different ratios between Fe-Si_3_N_4_ and Si_3_N_4_ powders were used. The compositions of the starting powders are shown in Table 1. The powders were balled milled in alcohol using a planetary mill for 24 h at a rotating speed of 300 rpm. After drying and sieving, the powders were pressed uniaxial in a stainless-steel die at a pressure of 70 MPa and were then cold-isostatically pressed at a pressure of 200 MPa. The sintering of the green bodies was conducted under a nitrogen pressure of 10 MPa at 1800 °C for 2 h.

The bulk density and the open porosity of the sintered specimens were measured according to the Archimedes principle and can be calculated from the equation:

(1) $$\rho =\frac{m_{1}}{m_{3}-m_{2}}\rho_{water}\;$$

(2) $$P=\frac{m_{3}-m_{1}}{m_{3}-m_{2}}\;$$

where *ρ* is the bulk density, *m*_1_ is the dry mass of the samples in air, *m*_2_ is the mass of the specimen when fully impregnated with the water, and *m*_3_ is the impregnated mass whilst suspended in the water. Phase compositions of the samples were identified by X-ray powder diffraction analysis (XRD, Rigaku-D/max-2400; Tokyo, Japan). Microstructures were observed by back scattered electron images (BSE, S-4700, Hitachi, Tokyo, Japan) on the polished surfaces so as to reveal the morphologies and distribution states of the *β*-Si_3_N_4_ grains, the grain boundary phases, and the Fe_3_Si particles. Image analysis was conducted to determine the phase content and the particle size with image analysis software that analyzed ten different back scattered electron (BSE) images. The microstructures of the fracture surfaces of the ceramics were observed using SEM (S-4700, Hitachi, Tokyo, Japan). The elemental composition was analyzed with an energy dispersive X-ray spectrometer (EDS). Indentations were placed on the polished surfaces by a Vickers indenter with a load of 9.8 N holding for 15 s to measure the hardness of the sintered samples, and then the indented surfaces were observed by SEM to examine the crack/microstructure interactions. Flexural strengths of the composites were tested by three-point bending on bars that were 40 mm long, 4 mm wide, and 3 mm thick according to the ASTM-D790 standard, using a 30 mm span and a crosshead speed of 0.5 mm/min. Fracture toughness was evaluated by single-edge notched beam (SENB) according to the ASTM-C1421-01b standard at a span of 20 mm and a crosshead speed of 0.05 mm/min using bar samples that were 30 mm long, 2 mm wide, and 4 mm thick. Fracture toughness was also calculated by the Vickers indentation technique through the following equation:

(3) $$K_{IC}\;=\;0.043H\sqrt{a}{\left(\frac{E\phi }{H}\right)}^{2/5}{\left(\frac{c}{a}\right)}^{-3/2}\;$$

where *K_IC_* is the fracture toughness, *H* is the hardness, *E* is Young’s modulus, *ϕ* is the constraint factor (~3), and *c* and *a* are half length of the diagonal of the indentation and the average crack length that was introduced by the indentation.

## 3. Results and Discussion

The density and open porosity of the Si_3_N_4_-Fe_3_Si composites are listed in Table 2. It can be found that the dense ceramic composites with high densities (≥3.2 g/cm^3^) and low open porosities (≤2.06%) were prepared successfully by gas-pressure sintering.

**Figure 2:**
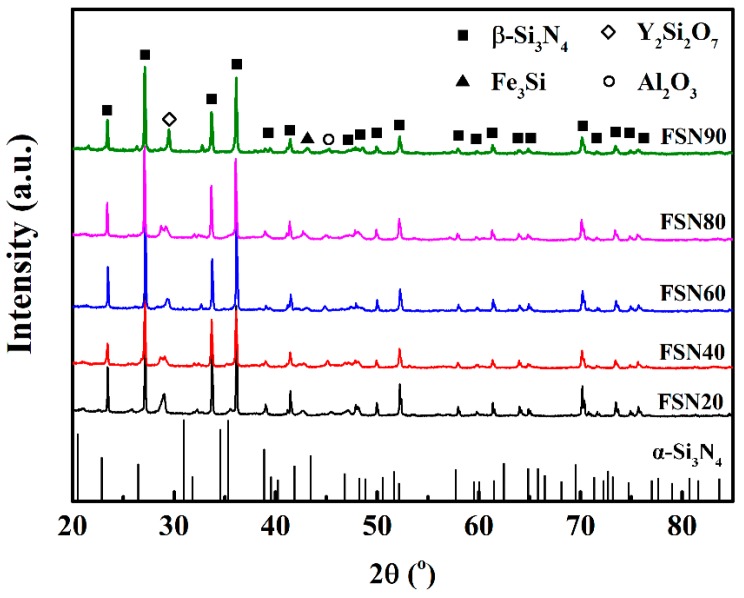
X-ray diffraction patterns of FSN20, FSN40, FSN60, FSN80, and FSN90. Peak positions of *α*-Si_3_N_4_ (ICDD PDF Card No. 41-0360) are denoted as the vertical lines at the bottom of the coordinate system.

Figure 2 shows the X-ray diffraction patterns of the sintered samples. The crystalline phases that were identified in these spectrums include *β*-Si_3_N_4_ (ICDD PDF Card No. 33-1160), Y_2_Si_2_O_7_ (ICDD PDF Card No. 38-0440), Fe_3_Si (ICDD PDF Card No. 35-0519), and Al_2_O_3_ (ICDD PDF Card No. 23-1009). It is obvious that in all of the samples, the main phases were *β*-Si_3_N_4_ and no *α*-Si_3_N_4_ could be detected, indicating a fully *α* → *β* phase transformation during the liquid phase sintering. Diffraction peaks around the 2θ of 29.4° indicate the existence of a Y_2_Si_2_O_7_ phase, which was formed at the grain junction of Si_3_N_4_ by the reaction between sintering aid Y_2_O_3_ and SiO_2_ at the surface of Si_3_N_4_ powder, which crystallized when it was cooled [31]. Studies have shown that the crystalline Y_2_Si_2_O_7_ secondary phase is beneficial for high temperature oxidation resistance and thermal mechanical properties of Si_3_N_4_ based materials [32,33]. Fe_3_Si was identified in all of the samples, suggesting that Fe_3_Si was stable at the sintering condition and was successfully introduced into the composites by adding Fe-Si_3_N_4_ to the raw materials.

It has been found that ferrous metals (Fe, Ni, Co, etc.) have a high affinity for Si, so that SiC and Si_3_N_4_ are reactive to Fe and some of its alloys [34,35]. T. Shimoo and K. Okamura [36] studied the reactions between silicides of Fe and Si_3_N_4_. In their work, they found that the silicides with a low Si/metal ratio react with Si_3_N_4_ to produce those with a high Si/metal ratio. At 1250 °C under Ar atmosphere, FeSi can be generated through the following overall reaction:

3Fe_3_Si + 2Si_3_N_4_ = 9FeSi + 4N_2_ (4)

**Figure 3:**
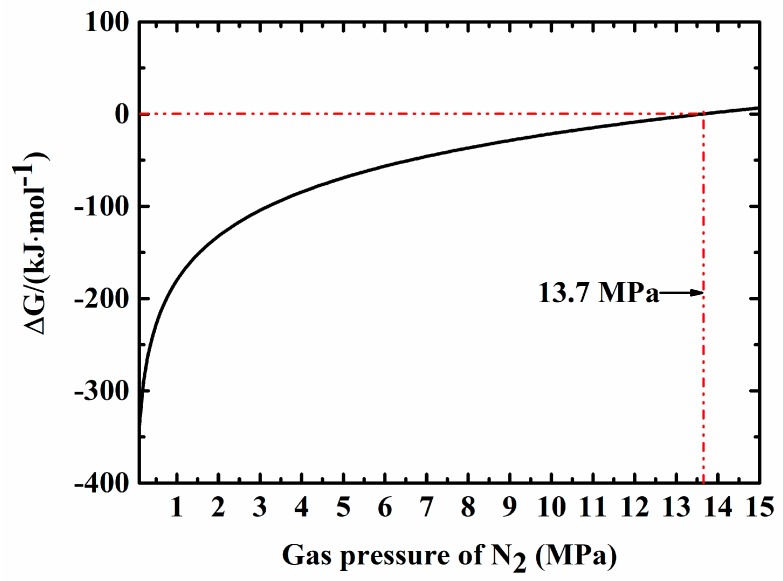
Gibbs free energy of reaction (4) at 1800 °C under different N_2_ pressures.

However, in our system, the sintering was conducted under a high N_2_ pressure (10 MPa). Furthermore, in the process of gas-pressure sintering, with an increasing temperature, the pores of the ceramic became closed pores so that the N_2_ that was produced through the above reaction could not be released, resulting in a local environment with an even higher N_2_ pressure which meant that the reaction could be suppressed, making Fe_3_Si stable to survive the sintering process. Thermodynamic analysis was done to calculate the Gibbs free energy of the reaction, and the results are present in Figure 3. It can be seen that with an increasing pressure of N_2_, the Gibbs free energy drops gradually. When N_2_ pressure was above 13.7 MPa, the Gibbs free energy was greater than 0 kJ·mol^−1^, indicating that the reaction could no longer continue. In our sintering condition, the external pressure of N_2_ was 10 MPa, therefore when the reaction occured and N_2_ was produced, the local pressure of N_2_ was much higher than 10 MPa, meaning the reaction could be suppressed or even stopped.

**Figure 4:**
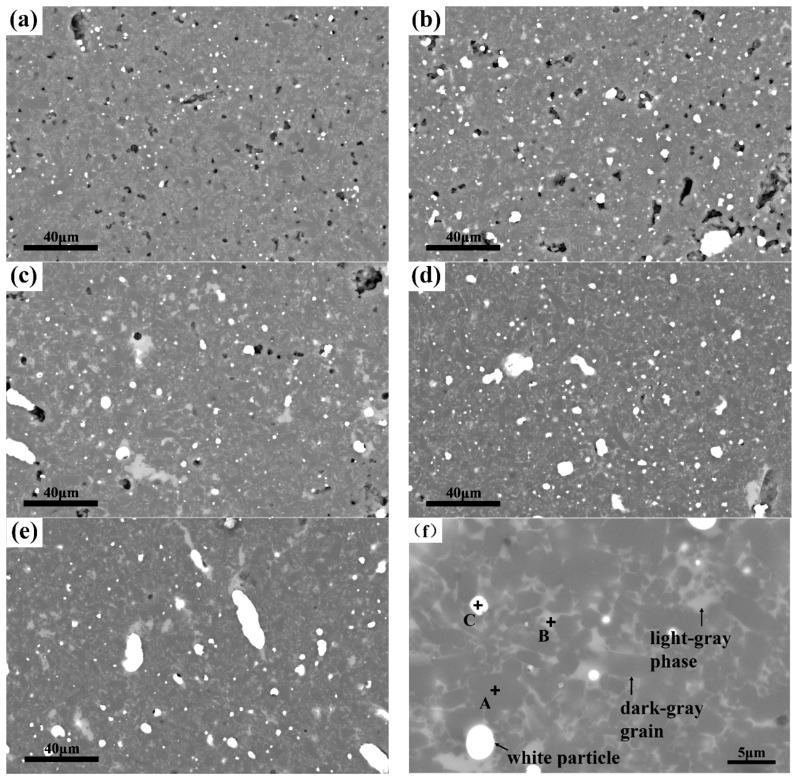
Back scattered electron (BSE) morphologies and (energy-dispersive X-ray spectroscopy) EDS analysis of the Si_3_N_4_-Fe_3_Si composites. (**a**–**e**) BSE morphologies of FSN20—FSN90. (**f**) High magnification of the BSE images and EDS analysis of FSN60.

**Figure 5:**
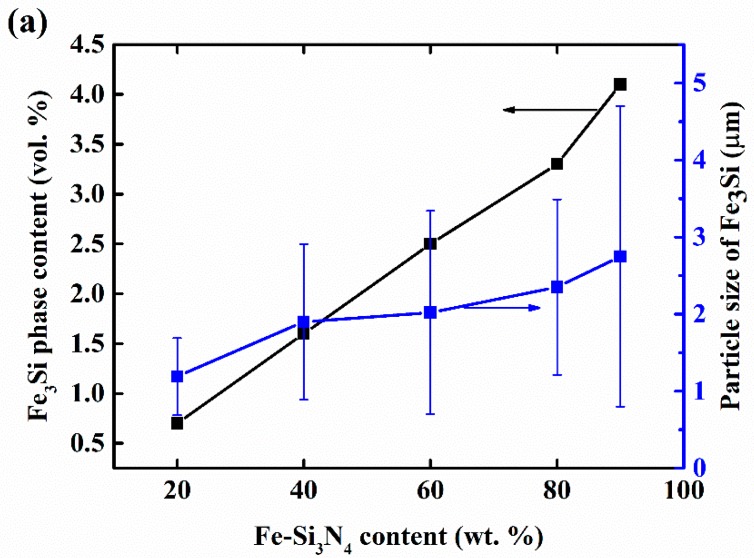

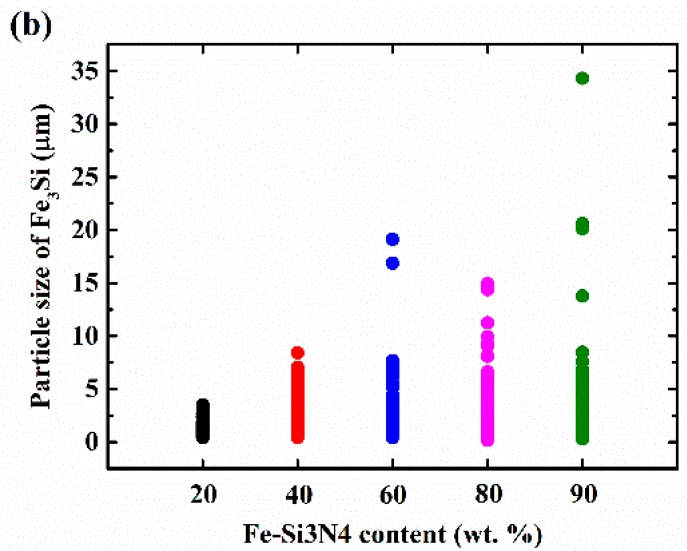
(**a**) Phase content, average particle size, and (**b**) particle size distribution of Fe_3_Si in the Si_3_N_4_-Fe_3_Si composites prepared with starting powders containing a different amount of Fe-Si_3_N_4_. In Figure 5b, 250 data of each particle size of each sample (represented by round dots with different colors) were selected randomly to illustrate the particle size distribution.

Figure 4 shows the BSE images and energy-dispersive X-ray spectroscopy (EDS) analysis of the composites. It can be seen from Figure 4a–f that all of the samples were mainly composed of three phases which are indicated by arrows in Figure 4f: the dark-gray columnar grains, the light-gray phases, and the white dispersive particles. EDS analysis (Table 3) confirmed that they are Si_3_N_4_, grain boundary phases, and Fe_3_Si, respectively. It can be clearly noted that, with the increase of Fe-Si_3_N_4_ content in the raw materials, the content of the Fe_3_Si phase in the composites arises. More importantly, the particle size of Fe_3_Si grows remarkably. In Figure 4a, the particle size of Fe_3_Si is smaller than 5 μm. However, in Figure 4e, Fe_3_Si particles that are bigger than 20 μm can be found. This phenomenon was confirmed by particle size measurements through image analysis (Figure 5). In Figure 5a, it can be found that the volume content rose in approximate linearity with the increase of Fe-Si_3_N_4_ content. The volume fraction of Fe_3_Si for FSN20, FSN40, FSN 60, FSN80, and FSN90 after sintering are 0.7, 1.6, 2.5, 3.3, and 4.1 vol. %. Assuming that Fe-Si_3_N_4_ contains 18 wt. % Fe_3_Si and the density of Fe_3_Si is 6.34 g/cm^3^ [37], the content of Fe_3_Si for each sample before sintering are calculated and the results (see Table 1) are 1.9, 3.9, 5.9, 8.1, and 9.2 vol. % respectively. So, we can estimate that about 60% of Fe_3_Si are sacrificed. There may be two reasons for the loss of Fe_3_Si. Firstly, Fe_3_Si dissolves into the grain boundary phase and EDS results showed that Fe exists in it. Secondly, since our method was based on the image analysis of BSE images, Fe_3_Si particles that were too small to recognize would have been neglected.

The average particle size of Fe_3_Si increased gradually from 1.19 μm to 2.75 μm. Figure 5b gives the particle size distribution of Fe_3_Si, from which it can be discovered that with the increase of Fe-Si_3_N_4_, although the average particle size of Fe_3_Si rose mildly, the abnormal growth of the particles occurred, and the number of big Fe_3_Si particles grew rapidly. In samples of FSN80 and FSN90, although the average size of Fe_3_Si remains relatively small, many Fe_3_Si particles that were bigger than 10 μm can be found frequently, indicating that with the increase of Fe-Si_3_N_4_ content in the starting powder, the degree of microstructure inhomogeneity would rise.

The difference in particle sizes and their distributions of Fe_3_Si in different samples can be explained by the flow of liquid Fe_3_Si in porous Si_3_N_4_ during the sintering process. Since the melting point of Fe_3_Si is about 1280 °C [38], at sintering temperature, the Fe_3_Si is in liquid state and can flow easily. When the content of Fe-Si_3_N_4_ was low and the Si_3_N_4_ content was high, the frameworks that were formed by the Si_3_N_4_ particles were relatively dense. Hence, the melting Fe_3_Si droplets were separated from each other and existed discretely. With the increase of the content of Fe-Si_3_N_4_ and the decrease of the content of Si_3_N_4_, the Si_3_N_4_ frameworks were weakened, and when the porosity of Si_3_N_4_ and the content of Fe_3_Si reached a critical point, percolation occurred and Fe_3_Si droplets joined each other and flowed to form bigger droplets and solidified into solid particles upon cooling.

**Figure 6:**
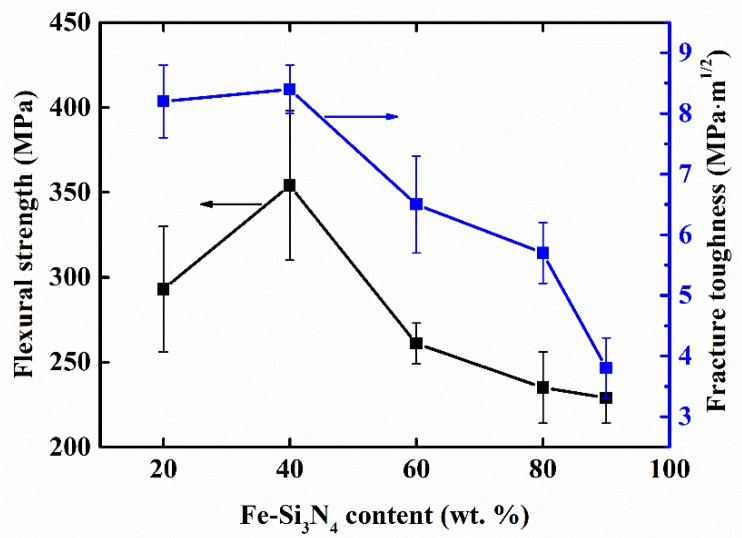
Flexural strength and fracture toughness of Si_3_N_4_-Fe_3_Si composites (FSN20, FSN40, FSN60, FSN80, and FSN90) prepared with starting powders containing different contents of Fe-Si_3_N_4_.

The mechanical properties of the Si_3_N_4_-Fe_3_Si composites are presented in Table 2 and Figure 6. From Table 2, we can see that the Vickers hardness of the samples decreased with the increase of Fe_3_Si content, which is as expected because the hardness of Fe_3_Si is much lower than that of Si_3_N_4_. Figure 6 shows the dependence of the flexural strength and fracture toughness of the Si_3_N_4_-Fe_3_Si composites on the content of Fe-Si_3_N_4_ in starting powders. It can be seen that the flexural strength and fracture toughness of FSN20 are 293 MPa and 7.9 MPa·m^1/2^, respectively. The highest flexural strength and fracture toughness were obtained (354 MPa and 8.4 MPa·m^1/2^) when the content of Fe-Si_3_N_4_ increased to 40 wt. %. However, a further increase in Fe-Si_3_N_4_ resulted in a gradual degradation of the mechanical properties of the composites. The above results showed that by carefully adjusting the composition of the raw materials, a Si_3_N_4_-Fe_3_Si composite with improved mechanical properties can be obtained. Dense monolithic Si_3_N_4_ ceramics that are fabricated using Si_3_N_4_ powder with high purity typically show good mechanical properties (three point bending strength of 400~900 MPa and fracture toughness of 3.4~8.2 MPa·m^1/2^ [39]). Our results show that by replacing 40 wt. % Si_3_N_4_ powder with the cost-effective Fe-Si_3_N_4_ powder, composites can obtain mechanical properties—especially fracture toughness that is at the same level with dense monolithic Si_3_N_4_ ceramics.

**Figure 7:**
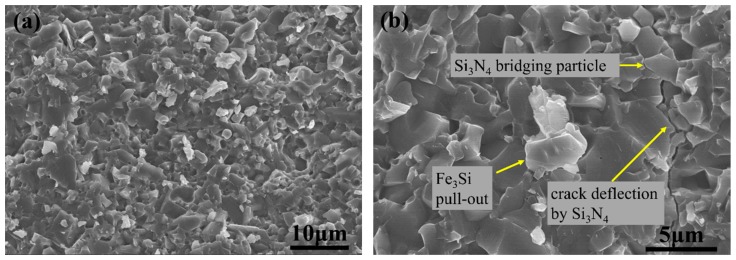
Fracture surface of FSN40. (**a**) Low magnification. (**b**) High magnification.

In order to research the microstructure—the mechanical properties’ relationship of the composites, fracture surface and crack/microstructure interactions were observed. Figure 7 shows the fracture surface of FSN40. In Figure 7a, it can be seen that fracture modes of the composites were in co-action by transgranular and intergranular fracture. Since Si_3_N_4_ has its well-known characteristic of self-reinforcement, two kinds of typical toughening mechanisms of crack bridging and crack deflection by Si_3_N_4_ can be found in Figure 7b, which resulted from the elongated Si_3_N_4_ crystals that were bounded by the weak interface [40]. Apart from the toughening mechanisms that were caused by Si_3_N_4_, pull out of Fe_3_Si particle can also be found in Figure 7b, indicating that Fe_3_Si also plays a part in toughening the composites.

**Figure 8:**
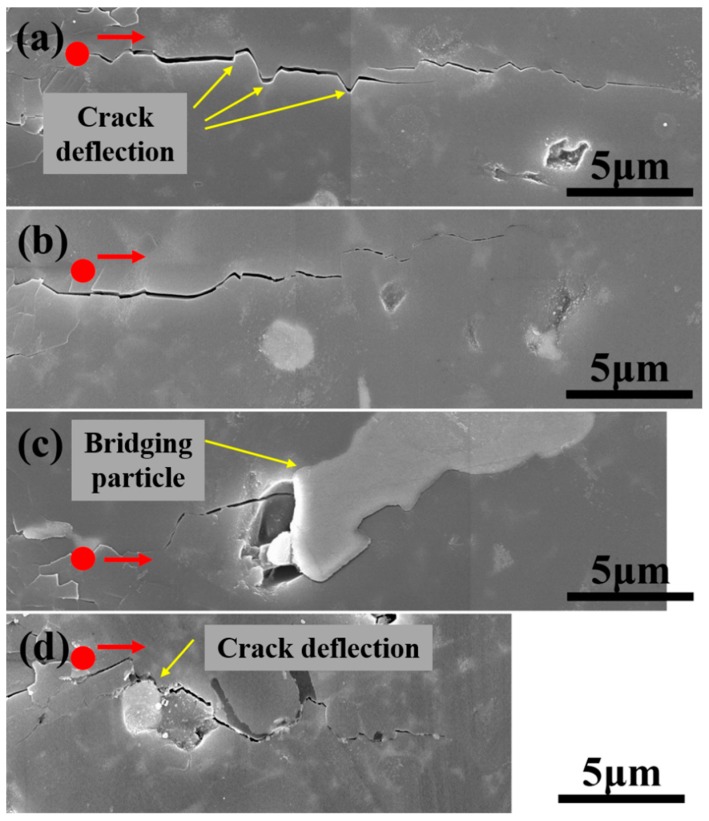
Crack/microstructure interactions in FSN60. Four typical cracks (**a**–**d**) were selected for detailed analysis. Red points stand for the diagonal apexes of the indentations. Red arrows stand for the propagation direction of the cracks.

The toughening effect of Fe_3_Si was further studied by Vickers indentation (Figure 8). Four typical cracks (Figure 8a–d) in sample FSN60 were selected for detailed analysis. The propagation paths of the cracks shown in Figure 8a,b were free of Fe_3_Si particles, and although crack deflection by Si_3_N_4_ crystals can be seen (Figure 8a), the crack lengths was relatively long (24.0 μm and 18.9 μm, respectively). In Figure 8c,d, where the cracks interacted with Fe_3_Si in the form of interface debonding, particle bridging, and crack deflection, the crack length was smaller (15.3 μm and 14.7 μm, respectively), which indicates an improved toughness. Since Fe_3_Si has better plasticity than the brittle Si_3_N_4_, when the crack propagated to the vicinity of the Fe_3_Si particle, the stress concentration around the crack tip can be somewhat reduced, thus the tendency to the ripping of the material was inhibited. The CTE of Fe_3_Si (14.4 × 10^−6^ K^−1^ [41]) was much bigger than that of Si_3_N_4_ (2.9 × 10^−6^ K^−1^ [39]), so the interface between Fe_3_Si and Si_3_N_4_ was under tensile stress at room temperature, resulting in a weak interfacial bonding strength. When under stress, the weak interface between Fe_3_Si and the surrounding phase debonded (Figure 8c,d). This led to a tortuous crack path (Figure 8d) or particle bridging (Figure 8d).

With the above-revealed toughening mechanism of Fe_3_Si, the dependence of the flexural strength and fracture toughness of the Si_3_N_4_-Fe_3_Si composites on the content of Fe-Si_3_N_4_ in starting powders can be explained. The inherent brittleness of ceramics is determined by its poor plasticity, so that when under stress, little energy can be consumed by the plastic flow. In order to improve the fracture toughness of ceramics, other energy dissipation mechanisms like crack deflection, bridging, or particle pull-out are often utilized in ceramic composites. Compared with the sample of FSN20, FSN40 contains more Fe_3_Si particles and the particle size of Fe_3_Si in it remains small (Figure 5). Large amounts of fine dispersed Fe_3_Si particles improved the strength and toughness of FSN40. When the content of Fe-Si_3_N_4_ in starting powders was more than 60 wt. %, despite the fact that the phase content of Fe_3_Si increased, the Fe_3_Si particles suffered severe growth, and large particles that were bigger than 15 μm emerged (Figure 5b). Large Fe_3_Si particles result in a non-uniform microstructure and may serve as crack origins when the samples are loaded, so the mechanical properties of FSN60, FSN80, and FSN90 are damaged. Narciso [42,43] studied the coefficient of the thermal expansion (CTE) properties of several metal-ceramic composites, and it was found that metals usually have CTE one magnitude higher than that of ceramics. In our study, the interface of Fe_3_Si and Si_3_N_4_ are under residue tensile stress due to the mismatch of CTEs. The residue tensile stress may result in cracks and voids between Fe_3_Si and Si_3_N_4_, which can act as a crack origin when under stress, and this may be one reason for the low mechanical properties of the composite with a high Fe_3_Si content.

## 4. Conclusions

In this study, Si_3_N_4_-Fe_3_Si composites with a different content of the Fe_3_Si phase were fabricated using starting powders of different compositions. The microstructure and mechanical properties of the composites were investigated by various methods. Special attention was placed on the particle size distribution of Fe_3_Si and their effect on the mechanical properties of the composites. The main conclusions can be summarized as follows.

The sintered composites were mainly composed of Si_3_N_4_, Fe_3_Si, and the grain boundary phase. With the increase of Fe-Si_3_N_4_ powder from 20 wt. % to 90 wt. % in the starting powder, the Fe_3_Si phase content increased from 0.7 vol. % to 4.1 vol. %, and the particle size increased from 1.2 μm to 2.8 μm. When more than 60 wt. % Fe-Si_3_N_4_ was added to the starting powders, the abnormal growth of Fe_3_Si particles occurred and particles bigger than 15 μm were commonly seen, leading to non-uniform microstructures and poor mechanical properties. The dispersive Fe_3_Si particles had a toughening effect on the composites through mechanisms such as particle pull-out, interface debonding, crack deflection, and particle bridging. Owing to the uniform microstructure and Fe_3_Si toughening, FSN40 showed the highest flexural strength and fracture toughness of 354 MPa and 8.4 MPa·m^1/2^, respectively, indicating great potential as thermal structural materials.

## Figures and Tables

**Table 1:** Compositions of the starting powders used to prepare Si_3_N_4_-Fe_3_Si composites.

Samples	Fe-Si_3_N_4_	Si_3_N_4_	Y_2_O_3_	Al_2_O_3_	Fe_3_Si in Starting Materials
	(wt. %)	(wt. %)	(wt. %)	(wt. %)	(vol. %)
FSN20	20	70	5	5	1.9
FSN40	40	50	5	5	3.9
FSN60	60	30	5	5	5.9
FSN80	80	10	5	5	8.1
FSN90	90	0	5	5	9.2

**Table 2:** Density, open porosity, and mechanical property of the Si_3_N_4_-Fe_3_Si composites.

Samples	Density (g/cm^3^)	Open Porosity (%)	Vickers Hardness (GPa)	Flexural Strength (MPa)	Fracture Toughness (MPa·m^1/2^)	
					SENB	Indentation
FSN-20	3.20	0.67	11.21	293 ± 37	8.2 ± 0.6	9.6 ± 0.9
FSN-40	3.28	0.46	9.91	354 ± 44	8.4 ± 0.4	10.1 ± 0.8
FSN-60	3.28	0.65	9.43	261 ± 12	6.5 ± 0.8	8.1 ± 0.8
FSN-80	3.29	2.06	9.19	235 ± 21	5.7 ± 0.5	7.0 ± 0.5
FSN-90	3.37	1.17	8.79	229 ± 15	3.8 ± 0.5	4.6 ± 0.7

**Table 3:** EDS analysis of spots in Figure 4f.

Element		Spot A	Spot B	Spot C
Atom ratio (%)	Si	55.4	22.5	23.0
	N	44.6	18.0	0
	Y	-	8.1	0
	Al	-	11.0	0
	O	-	38.9	0
	Fe	-	1.5	77.0
